# *Lycium barbarum* Polysaccharide Ameliorates Heat-Stress-Induced Impairment of Primary Sertoli Cells and the Blood-Testis Barrier in Rat via Androgen Receptor and Akt Phosphorylation

**DOI:** 10.1155/2021/5574202

**Published:** 2021-05-03

**Authors:** Suqin Hu, Dianlong Liu, Sijia Liu, Chunrui Li, Jian Guo

**Affiliations:** Department of Physiology, School of Traditional Chinese Medicine, Beijing University of Chinese Medicine, Beijing 100029, China

## Abstract

Male infertility induced by heat stress has been attracting more and more attention. Heat stress not only causes apoptosis of spermatocytes but also has adverse effects on Sertoli cells, further damaging spermatogenesis. *Lycium barbarum* polysaccharide (LBP) is the main bioactive component of *Lycium barbarum*, which has a protective effect on male reproduction, but its mechanism is still unclear. In this study, our results proved that LBP blocked the inhibitory effect on the proliferation activity of Sertoli cells after heat stress, reversed the dedifferentiation of Sertoli cells induced by heat stress, and ameliorated the structural integrity of the blood-testis barrier. In addition, it increased the expression of the androgen receptor and activated Akt signaling pathway to resist heat-stress-induced injury of Sertoli cells.

## 1. Introduction

More than half of the childbearing couples could not have children due to male infertility [1, 2], which is induced by varieties of causes including the spermatogenic quantitative or qualitative defect, catheter obstruction or dysfunction, and hypothalamic-pituitary axis disorders [3, 4]. Among these situations, abnormal spermatogenesis is the primary culprit of impaired male fertility. Spermatogenesis is a temperature-dependent process [5, 6]. For most mammals, normal spermatogenesis entails the temperature in the scrotum to be lower than body temperature. After heat treatment to local testis, male animals display testicular damages, including local testis tissue hypoxia [7, 8], germ cells apoptosis [9], blood-testis barrier (BTB) dysfunction, and reduced sperm count and quality [10–12]. Thus, scrotal temperature increases and spermatogenesis is impaired, leading to male infertility.

Spermatogenesis depends on mature Sertoli cells (SCs). In the seminiferous tubule, SCs provide structural support and supply nutrients, functional proteins, and cytokines for spermatogenesis [13, 14]. The number of SCs is proportional to the number of germ cells [15]. Occludin and zonula occludens-1 exist between adjacent SCs [16] and compose BTB which provides a suitable microenvironment for spermatogenesis [17, 18]. The decrease of occludin and (or) zonula occludens-1 causes the damage of BTB integrity, negatively effecting spermatogenesis [15, 19].

Androgen receptor (AR) is a type I steroid receptor. Androgens have to bind to AR before they can regulate the development of germ cells and SCs. Expressed in SCs, an androgen receptor (AR) plays a crucial role in spermatogenesis [20, 21]. Studies demonstrated that the loss of AR directly influences the maturation and the final quantity of SCs [22, 23], as well as spermatogenesis [24]. Besides, the absence of AR causes increased permeability of BTB in vitro or in vivo [25, 26].

**Figure 1:**
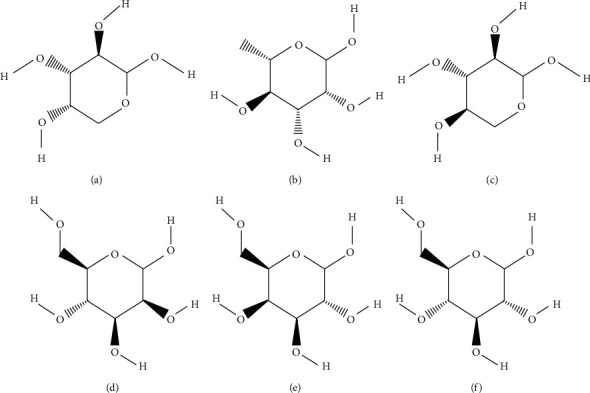
Six main monosaccharides in LBP. (a) Arabinose (PubChem CID: 439195); (b) rhamnose (PubChem CID: 25310); (c) xylose (PubChem CID: 135191); (d) mannose (PubChem CID: 18950); (e) galactose (PubChem CID: 6036); and (f) D-glucose (PubChem CID: 5793).

*Lycium barbarum* polysaccharide (LBP, PubChem SID: 134223164), the main component of Chinese wolfberry, is characterized by high bioactivity and significant content [27]. The glucoside apart, which is composed of arabinose, rhamnose, xylose, mannose, galactose, and glucose (Figure 1), accounts for more than 90% of the LBP mass [28]. Evidence shows LBP could ameliorate testicular damage by upregulating the testosterone level and reducing germ cell apoptosis [29–32]. However, limited research has been conducted on how LBP influences SCs and BTB. In the present study, we explore the effects and mechanisms of LBP on heat-stress-induced damage of SCs and BTB, centered around the changes in AR.

## 2. Materials and Methods

### 2.1. Drug and Reagents

*Lycium barbarum* polysaccharide powder was purchased from Nanjing Manhay Medical Technology (Nanjing, China, No. zhe B2-20090288-37).

DMEM/F12, 0.25% trypsin-ethylenediaminetetraacetic acid, collagenase IV, fetal bovine serum (FBS), and penicillin-streptomycin liquid were purchased from Gibco (BRL, Gaithersburg, MD, USA). 3-(4,5-Dimethylthiazol-2-yl)-2,5-diphenyltetrazolium bromide (MTT), dimethyl sulfoxide (DMSO), phosphate buffered saline (PBS), and the bicinchoninic acid (BCA) protein assay kit were purchased from Solarbio Life Sciences (Beijing, China). SP link detection kits (biotin-streptavidin HRP detection systems) and the diaminobenzidine kit were purchased from ZSGB-BIO (Beijing, China). The anti-Ki67 (ab16667) and anti-AR (ab133273) were purchased from Abcam (Cambridge, UK). The anti-occludin (PA5-20755) and anti-zonula occludens-1 (61-7300) were obtained from Thermo Fisher Scientific (Waltham, MA, USA). The anti-Phospho-Akt (Ser473) (#4060) and anti-Akt (#4691) were obtained from Cell Signaling Technology (Boston, MA, USA). The anti-CK-18 antibody (10830-1-AP) and anti-beta-actin antibody (60008-1-lg) were obtained from Proteintech (Chicago, IL, USA).

### 2.2. Animals

Mature male Sprague Dawley rats (8 weeks of age) were purchased from Huafukang (Beijing, China, No. SYXK Jing 2011-0024). These rats were treated and sacrificed according to the National Institutes of Health Guidelines for the Care and Use of Laboratory Animals (NIH Publication No. 85-23, revised 1985). All animal procedures were approved by the Laboratory Animal Welfare and Ethics Committee of the Beijing University of Chinese Medicine (BUCM-4-2017010805-010).

### 2.3. Primary SC Isolation, Culture, and Heat Treatment

Sertoli cell isolation was according to the previous method [33]. Briefly, the testicular tissue was obtained from the testis and washed twice in PBS precooled at 4°C. After fully centrifuged at 1000 rmp about 5 min, the sediment was treated with collagenase IV (0.5 mg/mL) for 5 min. After washing twice and certification for 5 min, the sedimentation was secondly digested with trypsin (0.05%) for 5 min. FBS in the same velum was added to stop digestion. Then, the sedimentation was filtrated through a 100-mesh filter and centrifugated at 1000 rmp for 5 min. The cells were washed twice with DMEM/F12, collected in the culture medium (DMEM plus F12 with 10% FBS and 1% penicillin-streptomycin), and cultured at 35°C in a CO_2_ incubator (5% CO_2_/95% air). After 40 h culturing, the medium was replaced to remove unattached germ cells, and 12–24 h later, when cells were confluent, they were ready for the following experiments. LBP was dissolved and diluted with PBS into different concentrations and then was added in SCs at the dose of 25 mg/L, 50 mg/L, and 100 mg/L. Cells in the control group and heat-stress group were added with the same volume of PBS. After 24 h, the control group was cultured at 35°C for another 20 minutes, and the other groups were treated in a 43°C water bath for 20 minutes.

### 2.4. Evaluation of Cell Viability

MTT assay has been widely used for measuring cell viability. The obtained absorbance value (OD value) under the specific wavelength is directly proportional to the number of living cells. SCs were seeded at 5 × 104 per well in 96-well plates in DMEM/F12 supplemented with 10% FBS. Cells were treated with LBP in different concentrations or PBS for 24 h. After removing some supernatant, an MTT regent (5 mg/ml) was added to each well for four hours. Then SCs were treated with DMSO by shaking for 15 min at room temperature, and the OD value was measured at 570 nm by using a microplate reader FLUO Star Omega (BMG Labtech, Offenburg, Germany). The blank group had no cells in wells and was used as the zero point of absorbance. Also, the absorbance value of each group divided by the control group was cell viability (%).

### 2.5. Immunohistochemistry

The immunohistochemistry protocol in our experiment followed that described previously [34]. SCs were first fixed in a 4% polyoxymethylene solution and then treated with 0.5% triton X-100 and 0.3% hydrogen peroxide, respectively. After three washes in PBS, cells were blocked with 10% goat serum to suppress the nonspecific antigen and then incubated in the primary antibody of Ki67 (1 : 200) or AR (1 : 200) overnight at 4°C. The next day, after three washes in PBS, the biotinylated secondary antibodies were added for 15 min at 37°C. After three washes in PBS, the horseradish enzyme labeling streptavidin working solution was added for 15 min at 37°C. Immunostaining was developed with the diaminobenzidine kit and counterstained with hematoxylin. Six nonoverlapping fields were selected for each group to take pictures. Image-Pro Plus (Version 6.0, Media Cybernetics, Bethesda, MD, USA) software was used to process the images and count the positive cells for statistical analysis.

### 2.6. Western Blot

Western blot was performed as described previously [35, 36]. The total protein was extracted from SCs and transferred to polyvinyl difluoride (PVDF) membranes. PVDF membranes were blocked in 5% nonfat milk for 1 h at room temperature and then exposed to the primary antibodies diluted in 1% blocking buffer: AR (1 : 1000), CK-18 (1 : 1000), occludin (1 : 1000), zonula occludens-1 (1 : 1000), p-Akt (Ser473) (1 : 1000), and Akt (1 : 1000) at 4°C overnight. After washing in tris-buffered saline containing 0.1% Tween-20 (TBST) three times, the membranes were incubated in horseradish-peroxidase- (HRP-) conjugated second antibodies (1 : 4000) for 1 h. After washing with TBST, the membranes were visualized by a hypersensitive electrogenerated chemiluminescence solution (Proteintech). *β*-Actin (1 : 5000) was used as an internal control for AR, CK-18, occluding, and zonula occludens-1. Akt was used as an internal control for phosphorylated-Akt in Ser473. Band intensities were determined by the software Quantity One, Version 4.6.2 (Bio-Rad Laboratories, Hercules, CA, USA).

### 2.7. Statistical Analysis

Each experiment was repeated at least three times. Statistical analysis was performed with the software SPSS version 20.0 (IBM, Albuquerque, NY, USA). Data that conformed to normal distribution or approximate normal distribution are expressed as means ± standard error of the mean (SEM). One-way analysis of variance was used for analyzing the data in different groups, and the pairwise comparisons were tested by the Tukey multiple comparison test. *P* < 0.05 was considered as significant, and *P* < 0.01 was considered as highly significant.

## 3. Results

### 3.1. The Changes in Cell Viability of SCs after Different Concentrations of LBP Treatment

**Figure 2:**
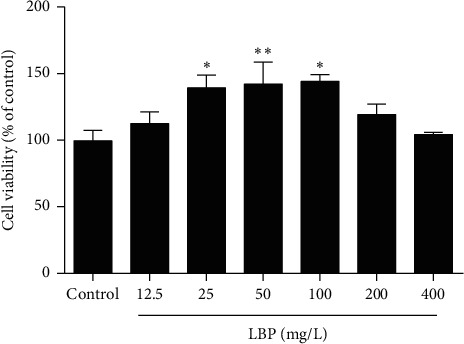
The changes of cell viability of SCs after different concentrations of LBP treatment. After treatment of LBP at 25 mg/L, 50 mg/L, and 100 mg/L, the cell viability of SCs was obviously increased. Data are presented as means ± SEM (*n* = 3) from three independent experiments. ^*∗*^*p* < 0.05 and ^∗∗^*p* < 0.01, compared to the control group.

To observe the effect of LPB on cell viability and select appropriate drug concentrations, we detected the OD value by MTT. As shown in Figure 2, compared with the control group, the cell viability of SCs at 25 mg/L, 50 mg/L, and 100 mg/L LBP treatment groups significantly increased (*P* < 0.05 or *P* < 0.01), while the differences in other LBP groups were not statistically significant (*P* > 0.05). Therefore, these three concentrations were selected as the concentration of LBP drug groups.

### 3.2. LBP Alleviates the Decrease of SC Proliferation Activity after Heat Stress

**Figure 3:**
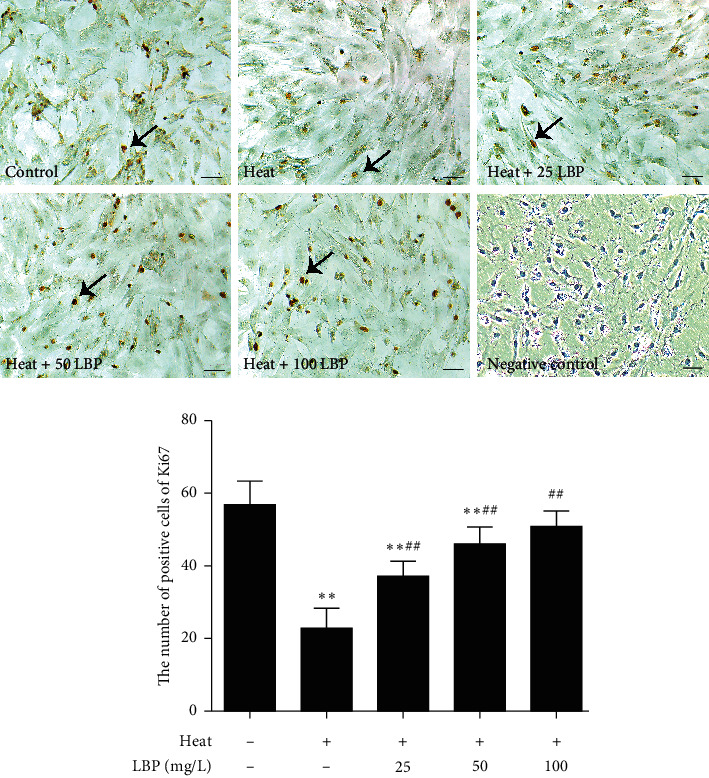
LBP improves the proliferation activity of SCs after heat stress. The expression of Ki67 was observed with the immunohistochemical assay. Scale bar: 50 *μ*m. The brown areas are Ki67 positive Sertoli cells (black arrows). Data are presented as means ± SEM (*n* = 3) from three independent experiments. ^∗∗^*p* < 0.01, compared to the control group. ^##^*p* < 0.01, compared to the heat-stress group.

To investigate the effect of LBP on the proliferation activity of SCs after heat stress, we tested the Ki67 in SCs (Figure 3). Ki67 expresses in the SCs nucleus. Compared with the control group, the positive signal of Ki67 significantly decreased in the heat-stress group and 25 mg/L LBP group (*P* < 0.05 or *P* < 0.01), and the 50 mg/L LBP and 100 mg/L LBP group have no significant change. Compared with the heat-stress group, the positive signal of Ki67 in LBP treatment groups showed a noticeable increase (*P* < 0.01). These data indicated that LBP treatment could resist the reduction of Ki67 expression induced by heat stress; however, the 25 mg/L LBP group still had a noticeable difference compared to the control group, and 50 mg/L LBP and 100 mg/L LBP could improve the proliferation activity of SCs after heat stress to the level of normal statement.

### 3.3. LBP Inhibits the Dedifferentiation of SCs after Heat Stress

**Figure 4:**
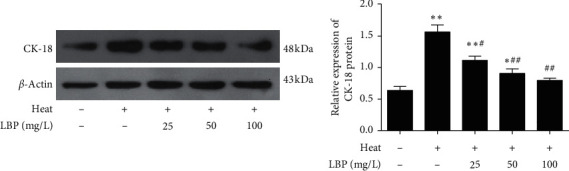
LBP inhibits the dedifferentiation of SCs after heat stress. CK-18 was checked by western blot. Data are presented as means ± SEM (*n* = 3) from three independent experiments. ^∗^*p* < 0.05 and ^∗∗^*p* < 0.01, compared to control group. ^#^*p* < 0.05 and ^##^*p* < 0.01, compared to the heat-stress group.

To observe the effect of LBP on the differentiation of SCs, we tested the expression of CK-18 (Figure 4). The expression of CK-18 in the heat-stress group significantly increased (*P* < 0.05 or *P* < 0.01) when compared with the control group. Moreover, compared with the heat-stress group, the expression of CK-18 in LBP treatment groups (25, 50, and 100 mg/L) decreased significantly (*P* < 0.01).

### 3.4. LBP Maintains the Integrity of BTB after Heat Stress

**Figure 5:**
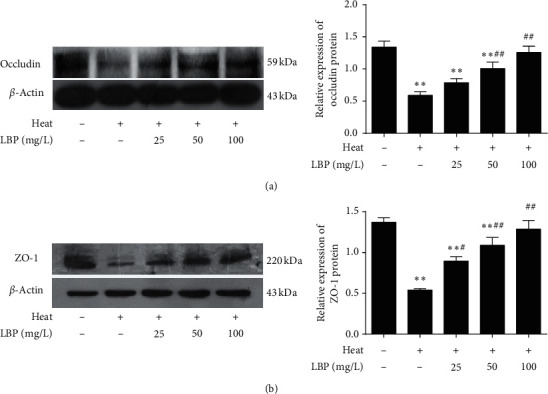
LBP maintains the integrity of BTB after heat stress. Occludin and ZO-1 in SCs were detected with western blot (a, b). Data are presented as means ± SEM (*n* = 3) from three independent experiments. ^∗∗^*p* < 0.01, compared to the control group. ^#^*p* < 0.05, ^##^*p* < 0.01, compared to the heat-stress group.

To clarify the effect of LBP on BTB, we detected the TJ-associated protein, occludin (Figure 5(a)) and zonula occludens-1 (Figure 5(b)). As shown in Figure 5, compared with the control group, the expression of occludin and zonula occludens-1 in the heat-stress group significantly decreased (*P* < 0.05 or *P* < 0.01). Compared with the heat-stress group, the expression of occludin and zonula occludens-1 in LBP treatment groups increased significantly (*P* < 0.01) in a dose-dependent manner (*P* < 0.05 or *P* < 0.01).

### 3.5. LBP Maintains the Expression of AR in SCs after Heat Stress

**Figure 6:**
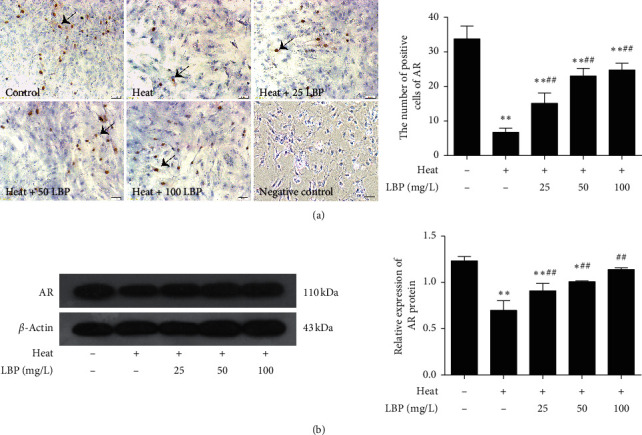
LBP upregulates the expression of AR in SCs after heat stress. The expression of AR was observed by immunohistochemical assay (scale bar: 50 *μ*m) and western blot (b). The brown areas are AR-positive Sertoli cells (black arrows). Data are presented as means ± SEM (*n* = 3) from three independent experiments. ^∗^*p* < 0.05 and ^∗∗^*p* < 0.01, compared to the control group. ^##^*p* < 0.01, compared to the heatstress group.

Testosterone only works when combining to the androgen receptor. To determine the AR in SCs and to better understand the mechanism of AR, we analyzed AR with immunohistochemistry staining (Figure 6(a)) and western blot (Figure 6(b)). Figure 6(a) shows that AR mainly expresses in the nucleus and few are expressed in the cytoplasm of mature SCs. As shown in Figure 6, the expression of AR did significantly decrease (*P* < 0.01) after heat treatment. Compared with the heat-stress group, the expression of AR in LBP treatment groups increased significantly (*P* < 0.01).

### 3.6. LBP Maintains the Akt Phosphorylation in SCs after Heat Stress

**Figure 7:**
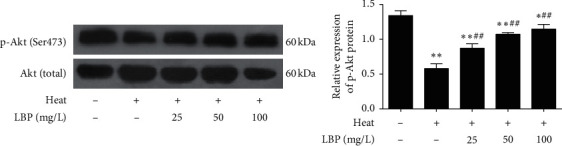
LBP promotes the Akt phosphorylation in SCs after heat stress. The phosphorylated Akt was detected with western blot. Data are presented as means ± SEM (*n* = 3) from three independent experiments. ^∗^*p* < 0.05 and ^∗∗^*p* < 0.01, compared to the control group. ^##^*p* < 0.01, compared to the heat-stress group.

We all know the critical role of the Akt signaling pathway in cell activity. It has been found that there is an interaction between AR and Akt phosphorylation at Ser473 [37]. Consequently, we tested the expression of p-Akt (Ser473) to explore the effective way of LBP on SCs (Figure 7). Compared with the control group, the expression of p-Akt (Ser473) in the heat-stress group significantly decreased (*P* < 0.01). Compared with the heat-stress group, the expression of p-Akt (Ser473) in LBP treatment groups increased significantly (*P* < 0.01). These data indicated that Akt phosphorylation at Ser473 was involved in the protective effect of LBP on SCs and BTB.

## 4. Discussion

Sertoli cells are the most crucial somatic cells for spermatogenesis. The number and maturations of SCs determine the spermatogenesis. In this study, our data indicate that LBP could resist the decrease of proliferation activity, inhibit the dedifferentiation of SCs after heat stress, and more importantly, preserve BTB integrity and permeability by maintaining AR and phosphorylated-Akt (Ser473).

Lycium barbarum fruits, as a traditional Chinese medicine and health food for people, have been used to nourish the kidney and improve fertility for thousands of years [38, 39]. The polysaccharide is the primary active component responsible for those biological activities in *L. barbarum* fruits [28]. Also, LBP has been reported to possess a wide range of pharmacological activities, including antioxidant, anticancer, and neuroprotective effects, immune regulation, and others [40]. Recently, there are some reports about the effects of LBP on male fertility, for example, increasing the serum testosterone level and decreasing apoptosis of germ cells. However, the effects of LBP on SCs and BTB are rarely reported. Thus, this study intends to investigate the effects and underlying mechanisms of LBP on SCs and BTB.

Temperature is an essential controller for reproductive activity and testicular homeostasis [41]. Only can physiological scrotal hypothermia guarantee the normal spermatogenesis in most mammals [42]. Despite that SCs are more tolerant to heat than germ cells, heat stress still can cause damaged structure and dysfunction of SCs [43], resulting in spermatogenic arrest and weak fertilizing capacity in vivo and in vitro [10, 44]. Some evidence and our previous study proved that local testis heat treatment (43°C for 20 min) successfully leads to dyszoospermia of monkey and rodents [33, 45, 46]. Furthermore, compared with other modeling methods of spermatogenesis disorder, heat-stress-induced impairment on testis is reversible, which is very suitable to study the sequence and interaction between SCs and germ cells.

We first selected three suitable concentrations of LBP by MTT (Figure 2) to treat Sertoli cell. As we previously said, the number of SCs directly determines the number of sperm. Ki67, a nuclear antigen closely related to cell mitosis, is often considered as a marker of cell proliferative activity [47, 48]. For rat SCs, the number of SCs keeps increasing after birth, as well as the number of Ki67 positive cells, while it began to decline at the age of 90 days [49], which suggest SCs of 60-day-old rats are not fully mature and some of them still can proliferate. Intervention on SCs of 60-day-old rats can affect the final number of SCs in seminiferous tubules. Therefore, we detected the expression of Ki67 to observe the proliferation activity of SCs. The result confirmed that LBP could preserve the cell proliferative activity of SCs after heat stress (Figure 3). Furthermore, only mature SCs can support spermatogenesis. CK-18 is a cytoskeleton molecule, which expresses in the prepubertal SCs and gradually disappears after puberty on mammals [50, 51]. High expression of CK-18 indicates that SCs are immature and dysfunctional [52]. Our results imply that LBP treatment could inhibit the re-expression of CK-18 after heat stress (Figure 4) to prevent the dedifferentiation of SCs.

The formation of BTB occurs at the beginning of puberty, and BTB is partly composed of tight junction (TJ), which directly affects the permeability [53]. Occludin is a highly phosphorylated transmembrane TJ-associated protein [54], believed as the initiator of BTB formation [55], and it is related to the initiation of spermatogenesis as well [56, 57]. Zonula occludens-1, a peripheral transmembrane protein, forms a link between the transmembrane proteins and the cytoskeletal compartment [58, 59] to maintain the integrity of BTB and support the migration and release of germ cells [60]. Reports have shown that increased permeability and dysfunction of BTB are blamed for the loss of occludin and (or) zonula occludens-1, resulting in harmful influence to spermatogenesis [61, 62]. Therefore, we selected occludin and zonula occludens-1 as the molecule marker of BTB integrity. Our data indicated that LBP could maintain the expression of them in SCs to ameliorate the heat-stress-induced increasing permeability and dysfunction of BTB (Figure 5).

It is well known that testosterone and AR are the decisive factors for maintaining male fertility and secondary sexual characteristics. AR is believed as a crucial upstream factor in controlling the development of SCs and the forming of BTB [63]. AR deficiency caused failure of SCs maturation [64] and decrease of TJ-associated protein expression [10, 11, 65]. In the present study, our data demonstrated that LBP could against the decrease of AR induced by heat stress, which was consistent with the change of tight junction protein, but opposite to CK-18 (Figure 6). Furthermore, through in vivo experiments, it was found that LBP increased the serum testosterone level in rats. We concluded the increase of testosterone and AR can improve their binding efficiency, which may be an important reason for LBP to ameliorate heat-stress-induced damage of SCs dedifferentiation and increase of BTB integrity and permeability.

Akt/protein kinase B (PKB), a serine/threonine kinase, is a mediator in the growth and proliferation of Sertoli cells [66, 67]. Akt is activated by phosphorylation on threonine 308 (Thr308) and serine 473 (Ser473), with phosphorylation at Ser473 resulting in maximal Akt activity [68]. Upregulated Akt phosphorylation could promote the expression of Ki67 [69]. Besides, studies showed that the Akt signaling pathway is related to the expressions of occludin and zonula occludens-1 [70, 71], and activation of Akt by enhancing phosphorylation of p-Akt (Thr308) and p-Akt (Ser473) can effectively prevent the destruction of the TJ barrier [72]. Also, after blocking the Akt signaling pathway, AR transduction was blocked into the testosterone signaling pathway in SCs, and Akt phosphorylation at Ser473 is the key molecule in the pathway of AR trafficking [37, 73]. In our results, the expression p-Akt (Ser473) of heat-stress SCs decreased while it increased after LBP treatment (Figure 7), as well as AR. Therefore, we concluded that the Akt signaling pathway involves in the effect of LBP on ameliorating heat-stress-induced damages in SCs and BTB.

**Figure 8:**
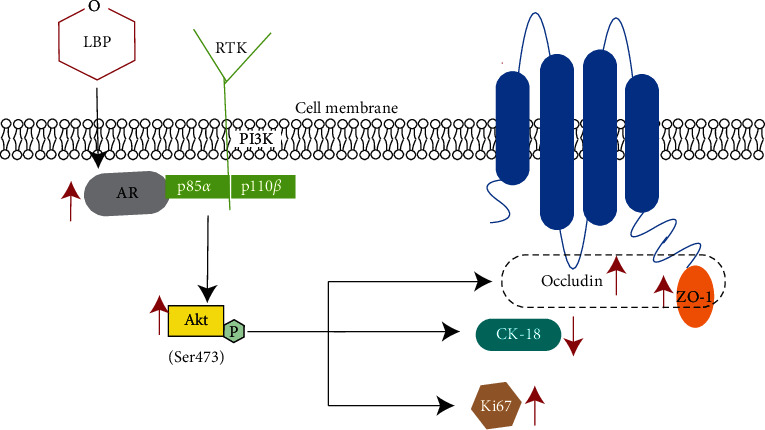
Effects and mechanism of LBP on Sertoli cells and BTB after heat stress. AR could directly interact with PI3K regulatory subunit p85*α* to activate kinase Akt [37, 73]. LBP maintained the expression of Ki67 and TJ protein and suppressed the re-expression of CK-18 by resisting the decrease of AR and maintaining phosphorylation of Akt in Sertoli cells after heat stress.

In summary, our study indicates that LBP can preserve the expression of Ki67 and occludin and zonula occludens-1 and inhibit the expression of CK-18 to prevent heat-stress-induced impairment of Sertoli cells and BTB through maintaining AR and Akt phosphorylation at Ser473 (Figure 8). Also, it provides the experimental evidence for clinical prevention of male reproductive heat-stress injury.

## Data Availability

The data supporting the conclusions of this article are included within the article.
